# Impaired Fellow Eye Motion Perception and Abnormal Binocular Function

**DOI:** 10.1167/iovs.19-26885

**Published:** 2019-08

**Authors:** Eileen E. Birch, Reed M. Jost, Yi-Zhong Wang, Krista R. Kelly, Deborah E. Giaschi

**Affiliations:** 1 Crystal Charity Ball Pediatric Vision Laboratory, Retina Foundation of the Southwest, Dallas, Texas, United States; 2 Department of Ophthalmology, UT Southwestern Medical Center, Dallas, Texas, United States; 3 Department of Ophthalmology and Visual Sciences, University of British Columbia, Vancouver, British Columbia, Canada

**Keywords:** amblyopia, binocular vision, motion perception

## Abstract

**Purpose:**

Binocular discordance due to strabismus, anisometropia, or both may result in not only monocular visual acuity deficits, but also in motion perception deficits. We determined the prevalence of fellow-eye deficits in motion-defined form (MDF) perception, the ability to identify a two-dimensional (2D) shape defined by motion rather than luminance contrast. We also examined the following: the causative role of reduced visual acuity and binocularity, associations with clinical and sensory factors, and effectiveness of binocular amblyopia treatment in alleviating deficits.

**Methods:**

Participants included 91 children with residual amblyopia (strabismic, anisometropic, or both; age, 9.0 ± 1.7 years), 79 nonamblyopic children with treated strabismus or anisometropia (age, 8.5 ± 2.1 years), and 20 controls (age, 8.6 ± 1.5 years). MDF coherence thresholds, visual acuity, stereoacuity, and interocular suppression were measured.

**Results:**

MDF deficits, relative to controls, were present in the fellow eye of 23% of children with residual amblyopia and 20% of nonamblyopic children. Stereoacuity and age first patched were correlated with MDF threshold (*r* = 0.29, 95% CI: 0.09–0.47; *r* = −0.33, 95% CI_:_ −0.13 to −0.50, respectively). MDF deficits were more common in children treated with patching alone than in those receiving contrast-rebalanced binocular treatment with games or movies (*t*_89_ = 3.46; *P* = 0.0008). The latter was associated with a reduction in mean fellow eye MDF threshold (*t*_26_ = 6.32, *P* < 0.0001).

**Conclusions:**

Fellow eye MDF deficits are common and likely reflect abnormalities in binocular cortical mechanisms that result from early discordant visual experience. Binocular amblyopia treatment, which is effective in improving amblyopic eye visual acuity, appears to provide a benefit for the fellow eye.

Binocularly discordant visual experience during early childhood can result not only in a monocular visual acuity deficit (amblyopia), but can also affect perception with the fellow eye, possibly due to abnormal development of binocular neurons that can be stimulated by either eye. Altered development may occur when childhood strabismus or anisometropia induce abnormal binocular interactions, interocular competition, and/or interocular suppression.^1^–^4^ Our understanding of the effects of binocularly discordant experience on fellow eye visual function is limited, and whether fellow eye deficits can be targeted to improve treatment outcomes remains to be determined.

One widely appreciated consequence of binocularly discordant visual experience is the subnormal or nil stereoacuity that typically accompanies amblyopia and often is resistant to rehabilitation by patching treatment.^5^–^7^ Measurement of stereoacuity does not allow the effects of discordant binocular visual experience on the amblyopic and fellow eyes to be examined separately; disparity detection inherently relies on comparison of inputs from both eyes. However, a subset of binocular cortical functions may provide insight into the effect of discordant binocular experience on the fellow eye, namely, binocular neurons that respond regardless of eye of origin.

Some neurons in V2^8^ and most neurons in MT^9^ respond to stimulation through either eye. In animal models of amblyopia these neurons show spiking irregularities,^10^ disrupted receptive field structure^8^ and abnormally high neuronal variability^9^ when driven by either the amblyopic eye or the fellow eye. These neuronal abnormalities correlate with the level of interocular suppression, as well as with the level of perceptual loss of contrast sensitivity or motion sensitivity in amblyopic monkeys, and they have been attributed to abnormal binocular interactions during early development.

The human homolog of MT, hMT/V5 has been implicated in the perception of motion-defined form^11^–^13^ (i.e., the ability to identify a two-dimensional [2D] shape defined by motion rather than luminance contrast).^14^–^18^ These neurons may not develop normally if they receive degraded information from one eye.^9^

Up to 40% of amblyopic children have been reported to have a fellow eye deficit in perceiving motion-defined form (MDF).^16^,^19^,^20^ It has also been reported that fellow eye MDF deficits were resistant to rehabilitation by patching.^16^ However, the sample sizes in these studies were small and included only amblyopic children and controls. As a result, it is unclear whether amblyopia or impaired binocular function was the primary causative factor for fellow eye MDF deficits. To address this limitation, three cohorts of children were evaluated: (1) amblyopic children, (2) nonamblyopic children who have been treated for strabismus, anisometropia, or both, and (3) control children. Amblyopic children have reduced visual acuity in the nonpreferred eye and impaired binocular function. Nonamblyopic children who have been treated for strabismus, anisometropia, or both have normal visual acuity but impaired binocular function. If the primary causative factor for fellow eye MDF deficits is amblyopia, amblyopic children should differ in prevalence and severity of MDF deficits compared with nonamblyopic children and controls. If the primary causative factor for fellow eye MDF deficits is impaired binocular function, both amblyopic and nonamblyopic children should differ in prevalence and severity of MDF deficits compared to controls.

This study had four aims: (1) determine the prevalence of fellow eye MDF deficits among amblyopic and nonamblyopic children treated for strabismus, anisometropia, or both, (2) determine whether reduced visual acuity (amblyopia) or reduced binocularity is the causative factor for fellow eye deficits, (3) investigate clinical and sensory factors that may be associated with risk for fellow eye MDF deficits in amblyopia, and (4) evaluate whether MDF deficits in amblyopic children can be ameliorated with binocular amblyopia treatment.

## Methods

### Participants

Ninety-one amblyopic children (7–12 years; 0.2–1.5 logMAR) with treated strabismus (*n* = 23), anisometropia (*n* = 41), or both (*n* = 33; combined mechanism) participated. Diagnostic criteria were those developed by the Pediatric Eye Disease Investigator Group for the Amblyopia Treatment Studies.^21^ Eligible amblyopic children had completed treatment with spectacles, patching, and/or binocular treatment but had residual amblyopia. Amblyopic eye best-corrected visual acuity (BCVA) was ≥0.2 logMAR, fellow eye BCVA was ≤0.1 logMAR, and interocular difference in BCVA was ≥0.2 logMAR. As a comparison group, age-similar nonamblyopic children with treated strabismus (*n* = 33), anisometropia (*n* = 29), or both (*n* = 18) were tested; 38 nonamblyopic children had no history of amblyopia or amblyopia treatment and 41 nonamblyopic children had recovered normal vision during prior treatment of amblyopia. BCVA was ≤0.1 logMAR in each eye, and interocular difference in BCVA was ≤0.1 logMAR. Age-similar controls were also enrolled (*n* = 20); BCVA was ≤0.1 logMAR in each eye, interocular difference in BCVA was ≤0.1 logMAR, and Randot Preschool stereoacuity was ≤1.8 log arcsec (≤60 arcsec). None of the children were born at <32 weeks postmenstrual age, had coexisting ocular or systemic disease, or had a history of congenital malformation or infection.

Written informed consent was obtained from a parent (and assent from children age ≥10 years) after explanation of the nature and possible consequences of the study. All procedures and the protocol were approved by the Institutional Review Board of University of Texas Southwestern Medical Center, followed the tenets of the Declaration of Helsinki and complied with the requirements of the US Health Insurance Portability and Accountability Act.

### Motion-Defined Form

In the MDF task, the child viewed (2.5 m viewing distance) a random array of white dots (1.7 arcmin^2^; 150 cd/m^2^) presented on a black background (11.5 × 6.6 deg; 1.0 cd/m^2^) with a dot density of 170 dots/deg^2^ and speed of 0.08 deg/s for 640 ms.^16^

**Figure 1:**
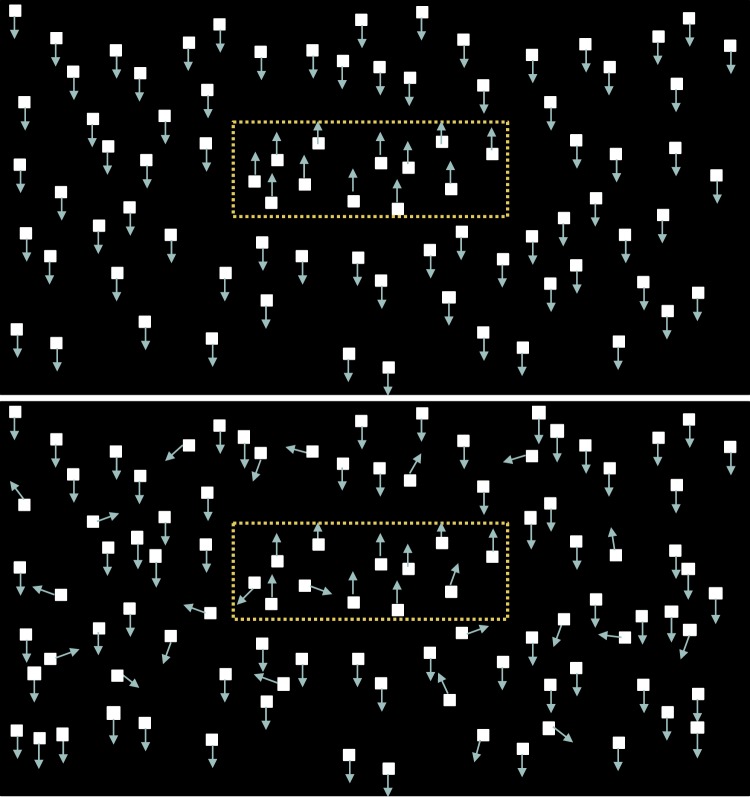
Top: MDF stimulus showing a horizontal rectangle defined by 100% coherently moving dots moving upward inside of a central horizontal rectangular area and 100% coherently moving dots moving downward outside of the rectangular area (i.e., a long spaceship). Bottom: The stimulus appearance when motion coherence is reduced to 75%; 25% of the dots are moving in random directions both inside and outside the rectangular area. Yellow dotted lines are not present on the display during testing; they have been added to the figure only to highlight the rectangular borders of motion contrast.

Dots within an invisible rectangle (2 × 1 deg) moved coherently in one direction (up or down), whereas dots outside of the rectangle moved in the opposite direction (Fig. 1). The context of the child's task was a Star Wars–like game. The child was asked to indicate the orientation of the “enemy spaceship” as “tall” or “long.” The proportion of coherently moving dots was progressively reduced from 100% in a 2-down 1-up staircase to determine the motion coherence threshold (i.e., the lowest % coherence at which the child could reliably discriminate the orientation of the rectangle). Children viewed a brief Powerpoint training presentation with instructions, followed by two practice trials, before proceeding to the MDF tests. For amblyopic children, the amblyopic eye was tested first, followed by the fellow eye. For nonamblyopic children, the formerly amblyopic eye was tested first for those who had a history of successfully treated amblyopia. For nonamblyopic children with no history of amblyopia, the right eye was tested. For controls, only the right eye was tested.

### Sensory Factors

BCVA was obtained for each eye with an opaque occluder patch and the ATS-HOTV (<7 years of age) or E-ETDRS (≥7 years of age) method using an EVA display system. Random dot stereoacuity was evaluated using the Randot Preschool Stereoacuity Test (Stereo Optical Co., Inc.; Chicago, IL, USA) and the Stereo Butterfly Test (Stereo Optical Co., Inc.), which were administered and scored according to manufacturer's instructions. Stereoacuity was reported as the finest disparity level passed on the Randot Preschool Stereoacuity Test (range: 1.3–2.9 log arcsec; 20–800 arcsec) or 3.3 log arsec (2000 arcsec) if unable to pass any level on the Randot Preschool Stereoacuity Test but passed the Stereo Butterfly Test, or nil if unable to pass either test. Depth of suppression was quantified by measuring the contrast balance index (CBI), which is the contrast ratio between dichoptically presented letters at which it is equally likely that the observer will report the left or right eye letter.^22^ CBI was calculated as the amblyopic eye contrast divided by the fellow eye contrast at this balance point.^22^,^23^

### Clinical Factors

Every amblyopic and nonamblyopic participant had a comprehensive eye examination by a pediatric ophthalmologist. Clinical factors, including etiology, age at which patching treatment was initiated, duration of patching treatment, and number of lines of visual acuity improvement with patching were obtained from medical records.

### Amblyopia Treatment

All amblyopic participants had received amblyopia treatment but still had residual amblyopia when their referring pediatric ophthalmologist decided to end treatment (other than spectacle correction) prior to enrollment in this study. Of the 91 amblyopic children 46 had been treated with patching. Typically, patching treatment was prescribed for 2 h/day 7 days/wk but, for some children at some time points, had been increased to 6 h/day. The other 45 children received binocular amblyopia treatment when they enrolled in a clinical trial (NCT02365090, NCT03288948, or NCT03825107). During the clinical trials, patching was discontinued. Children played contrast-rebalanced dichoptic games on a tablet 5 h/wk^6^,^24^ or watched contrast-rebalanced dichoptic animated videos 4.5 h/wk.^25^,^26^ Contrast-rebalancing of dichoptic games and videos (high amblyopic eye contrast and reduced fellow eye contrast) aims to decrease or eliminate suppression, promoting visual acuity recovery by providing binocular visual experience.^3^,^4^ Clinical trials of binocular amblyopia treatment lasted 2 to 8 weeks.

### Data Analysis

MDF thresholds from control children were used to establish 95% upper and lower tolerance limits, defined as the mean MDF threshold ±1.96 SD. Comparisons of MDF thresholds among amblyopic, nonamblyopic, and control children were conducted by 1-way ANOVA with pairwise comparisons by Tukey HSD tests. Associations between sensory factors and MDF thresholds and between clinical factors and MDF thresholds were evaluated by correlations (r and 95% confidence intervals for *r*). Comparisons of MDF thresholds among amblyopic children with strabismus, anisometropia, or both were conducted by 1-way ANOVA with pairwise comparisons by Tukey HSD tests. Paired *t*-tests were used for comparison of fellow eye thresholds between amblyopic children who had been patched and those who had binocular amblyopia treatment; fellow eye thresholds between amblyopic children before and after binocular amblyopia treatment; and MDF thresholds before and after binocular amblyopia treatment. Descriptive statistics for fellow eye deficits included calculating the percentage of children with fellow eye MDF deficits in subgroups of patients and the 95% confidence intervals for differences in proportions.

## Results

Baseline characteristics of the amblyopic, nonamblyopic, and control children are summarized in the Table. Mean age for amblyopic children was 9.0 ± 1.7 years, nonamblyopic children was 8.5 ± 2.1 years, and control children was 8.6 ± 1.5 years. On average, amblyopic children were patched for 2.4 ± 1.9 years before their pediatric ophthalmologist ended treatment. Mean improvement in BCVA with patching was 0.3 ± 0.3 logMAR (three lines). Forty-nine percent of amblyopic children also received 2 to 8 weeks of binocular treatment with contrast rebalanced dichoptic games or movies.^6^,^24^–^26^

**Table:** Clinical and Sensory Characteristics

	**Amblyopic,** ***n*** **= 91**	**Nonamblyopic,** ***n*** **= 79**	**Normal Control,** ***n*** **= 20**
% Female	45%	44%	45%
Age, y			
Mean ± SD	9.0 ± 1.7	8.5 ± 2.1	8.6 ± 1.5
Range	6.1–12.9	6.0–12.7	6.0–12.1
Diagnostic group, *n* (%)			
Strabismus	19 (21%)	33 (42%)	–
Anisometropia	41 (45%)	22 (28%)	
Both	31 (34%)	24 (30%)	
Amblyopic eye BCVA,* logMAR			
Mean ± SD	0.46 ± 0.26	–	–
Range	0.20–1.50		
Fellow eye BCVA,† logMAR			
Mean ± SD	−0.03 ± 0.07	−0.01 ± 0.08	−0.06 ± 0.07
Range	−0.10–0.10	−0.10–0.10	−0.10–0.10
Stereoacuity, log arcsecs			
Mean ± SD	3.50 ± 0.77	2.92 ± 1.08	1.60 ± 0.18
Range	1.60–nil	1.30–nil	1.30–1.80
Contrast balance index			
Mean ± SD	10.0 ± 16.7	3.8 ± 5.5	–
Range	0.9–99	0.8–32.3	
Age at initiation of patching, y			
Mean ± SD	4.6 ± 1.8	–	–
Range	0.5–6.3		
Duration of patching, y			–
Mean ± SD	2.3 ± 1.9	–	
Range‡	0.0–7.1		
BCVA improvement with patching, logMAR			–
Mean ± SD	0.30 ± 0.30	–	
Range	−0.30–1.20		
% with binocular treatment§	49%	–	–

* BCVA tested with ATS-HOTV (<7 years old) or E-ETDRS (≥7 years old).

† For normal controls, only the right eye was tested.

‡ Some children were treated only with contrast rebalanced dichoptic games or movies.

§ Binocular amblyopia treatment with contrast rebalanced dichoptic games^6^,^24^ or movies.^25^,^26^

### Fellow Eye MDF Deficits

**Figure 2:**
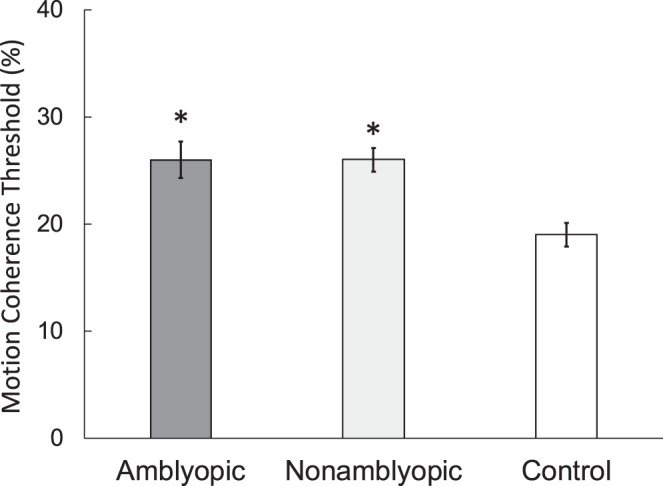
MDF thresholds (minimum % coherence that allowed for discrimination of rectangle orientation) for the fellow eyes of amblyopic children (n = 91; dark gray bar), one eye of nonamblyopic children (n = 79, 41 fellow eyes of successfully treated children and 38 right eyes of children with no history of amblyopia; light gray bar), and right eyes of control children (n = 20; white bar). *Group is significantly different from controls by Tukey HSD test.

Normal control children had a mean (±SD) right eye MDF threshold of 17 ± 5%; 95% tolerance limits for normal thresholds were 7% to 27% (Fig. 2). Overall, MDF deficits (threshold outside the 95% tolerance limits for age-similar control children) were present in 20 of 91 (23%) of fellow eyes of children with residual amblyopia and 16 of 79 (20%) of nonamblyopic children. There was a significant effect of group on fellow eye MDF threshold (*F*_2,189_ = 3.18, *P* = 0.04). Mean (±SD) fellow eye MDF thresholds for amblyopic children (26 ± 16%) and nonamblyopic children (26 ± 10%) were significantly worse than controls *P* = 0.03 and 0.02, respectively (Tukey HSD tests), but did not significantly differ from each other (*P* = 0.73). Nonamblyopic children had similar mean (±SD) fellow eye MDF thresholds whether they were never amblyopic (27 ± 13%; *n* = 38) or recovered with amblyopia treatment (23 ± 15%; *n* = 41).

### Amblyopia and Fellow Eye MDF

Of the three sensory indices of severity of amblyopia evaluated for the amblyopic children (amblyopic eye BCVA, stereoacuity, and suppression CBI), only stereoacuity was significantly correlated with MDF threshold (*r* = 0.29, 95% CI_:_ 0.09–0.47). Only 1 of 25 (4%) of amblyopic children with measurable stereoacuity had a fellow eye MDF deficit, whereas a significantly higher proportion of amblyopic children with nil stereoacuity had fellow eye MDF deficits (19 of 66; 29%; 95% CI for difference in proportions: 7%–37%). Interestingly, there was a similar pattern of prevalence among nonamblyopic children. Only 3 of 43 (7%) of nonamblyopic children with measurable stereoacuity had a fellow eye MDF deficit, whereas a significantly higher proportion of nonamblyopic children with nil stereoacuity had fellow eye deficits (13 of 36; 36%; 95% CI for difference in proportions: 11%–46%).

**Figure 3:**
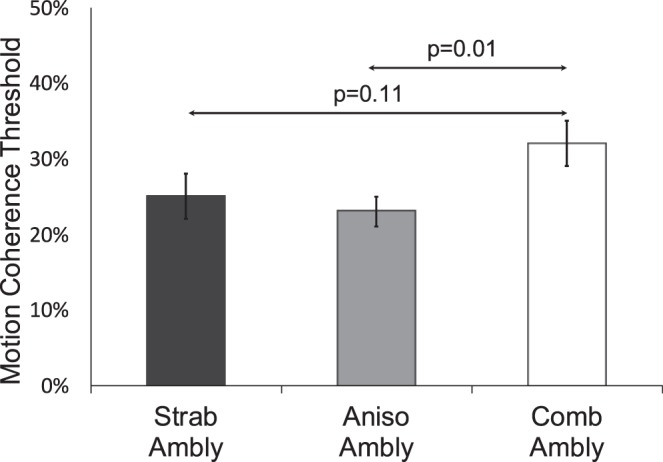
Fellow eye MDF thresholds (minimum % coherence that allowed for discrimination of rectangle orientation) for children with strabismic (n = 19), anisometropic (n = 41), and combined mechanism (n = 31) amblyopia.

The proportion of amblyopic children with a fellow eye MDF deficit was higher by a factor of 2 for combined mechanism amblyopia (10 of 30; 30%) compared with anisometropic amblyopia (6 of 41; 15%) or with strabismic amblyopia (4 of 23; 17%), but this difference was not statistically significant (95% CI for difference in proportions: 0%–38% and 0%–37%, respectively; Fig. 3). Mean (±SD) fellow eye MDF threshold was significantly elevated for combined mechanism amblyopia compared with anisometropic amblyopia (32% vs. 23%, *P* = 0.01). Of the three additional clinical factors examined (age first patched, duration of patching, lines of BCVA improvement with patching), only age first patched was linearly correlated with MDF threshold (age first patched: *r* = −0.33, 95% CI: −0.13 to −0.50; duration of patching: *r* = 0.16, 95% CI: −0.05 to 0.35; lines of BCVA improvement: *r* = −0.04, 95% CI: −0.24 to 0.17); the earlier the child started patching treatment, the more severe the fellow eye MDF deficit.

### Fellow Eye MDF Deficit and Treatment Regimen

**Figure 4:**
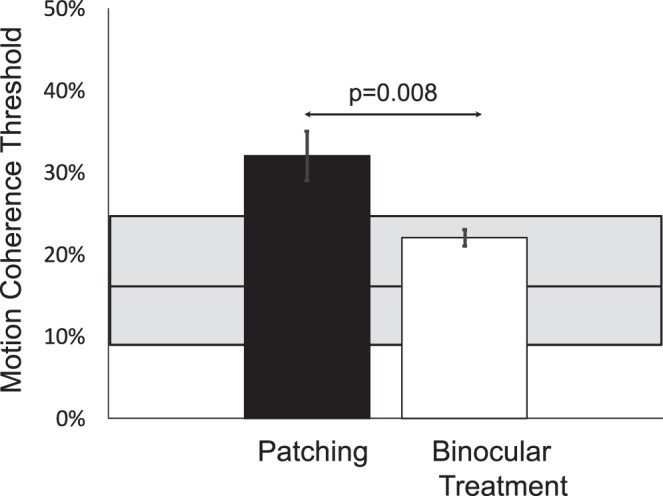
Fellow eye MDF thresholds (minimum % coherence that allowed for discrimination of rectangle orientation) for children who were treated with patching alone (n = 46) and children who received binocular treatment (n = 45). Gray shaded area shows the range of motion coherence thresholds observed in controls who received no treatment; the solid horizontal line shows the mean control threshold.

The cohort of 91 children with residual amblyopia included 46 children who had been treated with patching and 45 children who had binocular amblyopia treatment as either their sole treatment (*n* = 8) or in addition to patching treatment (*n* = 37; patching and binocular treatments in succession, not contemporaneous). Fellow eye MDF deficits were significantly more common among those treated with patching alone (14 of 46; 30%) than among those who had binocular amblyopia treatment (5 of 45; 11%, *z* = 2.27, *P* = 0.02) and more severe (mean ± SD MDF thresholds: 32 ± 20% vs. 21 ± 8%; *t*_89_ = 3.46; *P* = 0.0008; Fig. 4).

Twenty-seven of the 45 children who had binocular treatment had both a pretreatment and posttreatment MDF test available for analysis. Amblyopic eye BCVA improved from 0.51 ± 0.20 logMAR pretreatment (20/60) to 0.41 ± 0.25 logMAR posttreatment (*t*_26_ = 6.32, *P* < 0.0001). Mean fellow eye MDF threshold decreased from 29% to 19% (*P* = 0.0008). Pretreatment, 9 of 27 (33%) had an abnormal fellow eye MDF threshold and, posttreatment, only 2 of 27 (7%).

## Discussion

### Prevalence of Fellow Eye MDF Deficits

Fellow eye MDF deficits were common in 7- to 12-year-old children with residual amblyopia; 23% had fellow eye MDF deficits. This prevalence is similar to that previously reported for treated amblyopic children by Ho et al.,^19^ Hayward et al.,^20^ and Giaschi et al.^16^ In addition, control children in our study had mean (±SD) MDF threshold of 17 ± 5%, similar to that reported by Hayward et al.^20^ for 7- to 11-year-old controls, but better than the 38% reported by Giaschi et al.^16^ for a younger 4- to 8-year-old cohort of controls. Fellow eye MDF deficits were found among all types of amblyopia.

### Does Reduced Visual Acuity (Amblyopia) or Impaired Binocular Function Cause Fellow Eye MDF Deficits?

Both amblyopic and nonamblyopic children who have been treated for strabismus, anisometropia, or both were included in this study to investigate whether reduced visual acuity (amblyopia) or impaired binocular function causes fellow eye MDF deficits. If the primary causative factor for fellow eye MDF deficits was amblyopia, only amblyopic children should differ in prevalence and severity of MDF deficits compared with nonamblyopic children and controls. On the other hand, if the primary causative factor for fellow eye MDF deficits was impaired binocular function, we expected that both amblyopic and nonamblyopic children should differ in prevalence and severity of MDF deficits compared with controls. Consistent with the latter hypothesis, fellow eye MDF deficits were present in 20% of nonamblyopic children who had been treated for strabismus, anisometropia, or both, a prevalence similar to that found among children with residual amblyopia. Prevalence and severity of fellow eye MDF deficits in nonamblyopic children were similar for those who had never been amblyopic and those who had successfully treated amblyopia. Moreover, fellow eye MDF deficits were more common among both amblyopic and nonamblyopic children who had nil stereoacuity compared with those who had measurable stereoacuity. Neither amblyopic eye BCVA nor severity of suppression was correlated with fellow eye MDF threshold. Taken together, these results support the hypothesis that fellow eye MDF deficits likely reflect abnormalities in binocular cortical mechanisms as a result of early discordant visual experience.^18^

The neural locus of these deficits may start in V1 where signals from the amblyopic eye are weakened and binocularity is known to be disrupted,^27^ but changes in extrastriate visual areas are required to explain the full range of perceptual deficits in amblyopia,^28^,^29^ including those involving the fellow eye. Fellow eye abnormalities have been reported in monkey V2 and MT.^8^–^10^ The human lateral occipital complex and hMT/V5 have been implicated in the normal processing of MDF^12^; hMT/V5 shows less activation on motion perception tasks with amblyopic or fellow eye viewing.^30^,^31^ However, the neural basis of amblyopia is not yet well understood.

### Clinical and Sensory Factors Associated With Risk for Fellow Eye MDF Deficits

Age first patched also was associated with the severity of fellow eye MDF deficits; the earlier the child started patching treatment, the more severe the fellow eye MDF deficit. At first glance, this association might suggest that patching contributes to the fellow eye deficit. If that were the case, however, we might also expect that severity of fellow eye deficits would be associated with duration of patching and with the number of logMAR lines of BCVA improvement with patching (as a surrogate measure of patching compliance), but neither of these associations was observed in our data set. An alternative explanation of the association between age first patched and severity of the fellow eye deficit may be that congenital or early onset binocular deficits are associated with more severe fellow eye deficits. Later onset may not interfere with fellow eye MDF perception because this aspect of vision matures relatively early, even for the relatively slow dot speed used in the present study.^20^

### Can Fellow Eye MDF Deficits in Amblyopic Children Ameliorated With Binocular Amblyopia Treatment?

Fellow eye MDF deficits were significantly more common among those treated with patching than among those who had binocular amblyopia treatment. We were also able to observe that binocular amblyopia treatment was associated with a reduction in mean fellow eye MDF threshold and a higher proportion of children with fellow eye MDF thresholds within the normal range. These data support the effectiveness of binocular amblyopia treatment, designed to decrease or eliminate suppression and provide binocular visual experience,^3^,^4^ in rehabilitating fellow eye deficits.

### Limitations

This study had several limitations. Children were not randomized to patching versus binocular amblyopia treatment, and some children were patching prior to initiating binocular treatment. It is possible that children who received binocular amblyopia treatment differed in some way that was not captured by the variables summarized in the Table. Second, although age at diagnosis and age first patched were extracted from the medical records, we cannot be certain when amblyopia first developed. Third, although short-term binocular treatment was associated with a reduction in fellow eye MDF deficits, it is unknown whether a longer period of binocular treatment would yield continued improvement or resolution of the MDF deficit.
